# Does socioeconomic factors and healthcare coverage affect postpartum sterilization uptake in an urban, tertiary hospital?

**DOI:** 10.1186/s12978-023-01572-5

**Published:** 2023-01-27

**Authors:** Sutira Uaamnuichai, Phanupong Phutrakool, Nareerat Thammasitchai, Sarochinee Sathitloetsakun, Somsook Santibenchakul, Unnop Jaisamrarn

**Affiliations:** 1 Department of Obstetrics and Gynecology, Faculty of Medicine, Chulalongkorn University and King Chulalongkorn Memorial Hospital, 1873 Rama IV Road, Patum Wan, Bangkok, 10330 Thailand; 2 Chula Data Management Center, Faculty of Medicine, Chulalongkorn University, Bangkok, Thailand; 3 Nursing Department, King Chulalongkorn Memorial Hospital, Bangkok, Thailand; 4 Department of Obstetrics and Gynecology, Faculty of Medicine, Chulalongkorn University, Bangkok, Thailand

**Keywords:** Female sterilization, Health coverage, Contraceptives, Postpartum sterilization

## Abstract

**Background:**

Postpartum sterilization in Thailand has relatively few barriers compared to other countries. The procedure is covered by some healthcare plans, and paid out-of-pocket for others. We aim to determine if healthcare coverage and other socioeconomic factors affect the rate of postpartum sterilization in an urban, tertiary hospital.

**Methods:**

We conducted a secondary analysis of data from a retrospective cohort of 4482 postpartum women who delivered at our hospital. Multivariable logistic regression was conducted to determine if sterilization reimbursement affects immediate postpartum sterilization rate.

**Results:**

Overall immediate postpartum sterilization rate was 17.8%. Route of delivery and parity were similar in those who were reimbursed and those who were not. Women aged over 25 were more likely to have a healthcare plan that does not cover postpartum sterilization. Women whose healthcare plan reimbursed the procedure trended towards postpartum sterilization when compared to women who were not (aOR 1.05, 95% CI 0.86–1.28, p-value = 0.632). Women who delivered via cesarean section were more likely to undergo sterilization at the time of delivery (aOR = 5.87; 95% CI 4.77–7.24, p-value = < 0.001). Women aged 40–44 years were 2.70 times as likely to choose sterilization than those aged 20–24 years (aOR = 2.70; 95% CI 1.61–4.53, p-value < 0.001).

**Conclusions:**

Healthcare coverage of the procedure was not associated with increased postpartum sterilization in our setting.

## Plain Language Summary

Postpartum sterilization is an effective and popular method of contraception. In our hospital, postpartum sterilization is easily accessible compared to other countries where there are barriers such as mandatory waiting time, limited operating rooms and anesthesiologists. We examine factors that affect sterilization rates and found that older women and women with more children were more likely to choose postpartum sterilization. We also found that women who delivered by cesarean section were more likely to choose sterilization. Interestingly, whether the woman’s healthcare plan covers postpartum sterilization does not affect the likelihood of sterilization.

## Introduction

Postpartum sterilization is a safe and effective form of contraception [1]. Female sterilization was the most commonly used method of contraception in 2019, with 23.7% of women choosing it [2]. Sterilization rates vary widely in different countries: 25.2% of Thai women are sterilized as opposed to approximately 10% in other Asian countries, and approximately 60% in South Asia [2, 3]. A woman’s decision to be sterilized is complex and can be influenced by religion, education, superstition, and culture, among other factors [4]. Previous studies have shown that women with higher parity, older age, and less exposure to modern contraceptive methods were more likely to choose sterilization [3, 5]. Insurance coverage of the procedure is an important factor affecting the choice of contraception, including the mandatory waiting time and consent forms involved in some forms of insurance coverage [4, 6].

In the 1970s, Thailand made tremendous efforts to curb population growth by increasing education and access to contraception and sterilization [7]. Since then, the sterilization rate in Thailand has increased considerably, reaching a peak of 36% in the 1980s [8]. Studies on sterilization in Thailand have been sparse. Currently, postpartum sterilization is partially covered by different healthcare plans in Thailand, and government programs have recently been enforced to cover the use of oral contraceptive pills [9]; however, coverage is partial and different hospitals have different ways of implementing contraceptive coverage. Postpartum sterilization in Thailand is also relatively more accessible; there are no waiting times between decision and operation, and the informed consent and counseling process is relatively straightforward. The present study aimed to explore the effect of healthcare coverage of postpartum sterilization and other socioeconomic factors on sterilization rate in a tertiary hospital with readily available operating rooms and anesthesiologists.

## Materials and methods

### Study design and population

This study was a secondary analysis of a cross-sectional study conducted among postpartum women at a tertiary hospital in Bangkok, Thailand. The original study is a cross-sectional study describing postpartum contraceptive choices, immediately postpartum and at 6 weeks postpartum follow up visit, in women delivering before the COVID pandemic (January 1st to June 30th, 2019) and during the COVID pandemic (January 1st to June 30th, 2020). All women who delivered during the specified time period were included in the study. Women who delivered before 22 weeks (the limit of viability in our hospital), women with incomplete medical records, or women who underwent cesarean hysterectomy were excluded. The original cohort totaled 4506 women, 2352 from pre-pandemic and 2154 from the pandemic period. Demographic and obstetric data was collected from the medical records. Briefly, we found that there were no significant differences in demographic or obstetric characteristics between both groups. The rate of immediate postpartum contraception initiation was similar in both groups, including initiation of intra-uterine devices, contraceptive implants, and sterilization. At 6 weeks postpartum, there were no significant differences in contraceptive choice between the pre-pandemic and pandemic group.

The analysis reported in this study included all participants who delivered during the specified time period and had their immediate postpartum contraception choice recorded in the medical records. Women who delivered before 22 weeks, had incomplete medical records, underwent cesarean hysterectomy, or had no data on healthcare coverage were excluded.

### Study setting and background information

At our hospital, women receive contraceptive counseling during the third trimester of antenatal care and during the postpartum hospital stay by trained nurses. Choice of combined oral contraception (COC), progestin-only pills (POP), progestin-only injections, hormonal and copper intrauterine devices (LNG-IUS, Cu-IUD), contraceptive implants, male condoms, and postpartum and male sterilization is available, taking into account existing medical conditions and suitability. Contraceptive implants and Cu-IUDs is free of charge for adolescents and all women in Bangkok, where our hospital is located. Other methods of contraception are currently not covered by any healthcare plan. Contraceptive method of choice may be initiated immediately postpartum or at 6 weeks postpartum during follow up visit at the Family Planning Clinic. Thorough counseling and informed consent were obtained before initiating contraception.

Women may express sterilization intention during antenatal care, after delivery, or during postpartum hospital stay. Sterilization is available to all women without coercion and after thorough counseling on all contraceptive options along with risks and benefits of each method; sterilization is offered to women in their third or higher pregnancy and to high-risk pregnancies. If desired, sterilization is performed during cesarean delivery for both emergency and elective procedures. For women delivering vaginally, sterilization was performed prior to hospital discharge within two days of delivery, except women who delivered on Fridays, Saturdays, and Sundays who had to wait until Monday for sterilization. Spousal consent was not required, but the women were encouraged to inform their spouses about their decisions. The operating room and anesthesiologist were available on Mondays through Fridays for sterilization. Trained physicians performed sterilization under spinal anesthesia via mini-laparotomy. Patients who delivered vaginally were discharged on the second day post-delivery; those who were sterilized were discharged similarly, under these circumstances, patients could be discharged the following day. There were no mandatory waiting times between the expression of intent and the sterilization operations. All specimens removed from the patient during postpartum sterilization are histologically examined for complete resection. Patients undergoing both cesarean delivery and postpartum sterilization are assessed by the anesthesiologist team prior to operation.

Female sterilization is covered by healthcare plans to varying degrees. Universal coverage, government officers, and hospital officers were covered for all costs involved in the hospital stay, including female sterilization. Patients covered by social security were paid a lump sum of 15,000 baht (1 USD = 31.8–31.9 baht during the study period) to cover delivery and newborn costs, including costs of contraception; the patients could retain whatever remained from the lump sum. A small portion of patients who opted to deliver in a hospital outside their network paid out-of-pocket for hospital fees. Hospital fees at our hospital were generally 1300–4000 baht (35–108 USD) for vaginal delivery (an additional 8000 baht (217 USD) for postpartum sterilization), and 11,300 baht (300 USD) for cesarean delivery (with an additional 1100 baht (30 USD) for sterilization along with cesarean delivery). Generally, accommodation including meals for one day of hospital stay, costs 900–1800 baht (24–48 USD). To determine whether healthcare coverage affects the decision to be sterilized, we divided the analytic set into two groups: women who were covered for sterilization (universal coverage, hospital employees, and government officers) and women who were not fully reimbursed for sterilization (social security and out-of-pocket payment).

### Data extraction and data analysis

Demographic characteristics, obstetric characteristics, and contraceptive choice was extracted from electronic medical records. Demographic characteristics included age, ethnicity, marital status, and healthcare plan. Obstetric characteristics included parity, number of living children, route of delivery, obstetric complications, gestational age, and birthweight at delivery. Immediate postpartum contraception initiation was determined from discharge summary. Immediate postpartum contraception indicates that the contraceptive method of choice is initiated before hospital discharge. Postpartum sterilization was defined as sterilization within 6 weeks of delivery and interval sterilization as sterilization after 6 weeks from delivery.

Data were collected and managed using Research Electronic Data Capture (REDCap), a secure web-based software platform hosted at Chulalongkorn University [10]. Statistical analyses were performed using STATA version 17 (StataCorp. 2021. Stata Statistical Software: Release 17. College Station, TX: StataCorp LLC.). Quantitative variables were characterized by mean values with standard deviations (SD), and qualitative variables were characterized by the number and percentage of participants in each category. Multivariable logistic regression was used to determine the association between the different types of health coverage and postpartum female sterilization. Analyses were adjusted for age, parity, marital status, route of delivery, and period of delivery (pre- vs during pandemic).

## Results

We included 4482 women in the analysis. Their demographic characteristics are displayed in Table 1. The mean age of the population was 31.65 (*SD* = 5.54) years. Most (30%) participants were 30–34 years old and were married (69.7%). Forty-four percent were in their primiparous. Approximately half of the patients delivered via cesarean section and vaginally (51.1% and 48.9%, respectively). A majority of the women’s healthcare was covered by social security (62.7%), followed by universal coverage (20.3%), government and hospital officers’ reimbursement (7.5% and 3.1%, respectively); 6.4% paid out-of-pocket. Women were divided into those who were fully covered for postpartum sterilization (30.9%; universal coverage, government, and hospital officers) and those who were not (69.1%; social security and paid out-of-pocket).

**Table 1:** Demographic characteristics

	Total n (%)	Postpartum sterilization n (%)	Non-postpartum sterilization n (%)
*n (%)*	4482 (100)	797 (17.78)	3685 (82.22)
*Health care coverage*			
Social security	2810 (62.70)	478 (17.01)	2332 (82.99)
Self-paid	287 (6.40)	58 (20.21)	229 (79.79)
Universal coverage	908 (20.26)	171 (18.83)	737 (81.17)
Hospital officer	139 (3.10)	32 (23.02)	107 (76.98)
Government officer	338 (7.54)	58 (17.16)	280 (82.84)
*Reimbursement status*			
Not reimbursed for sterilization	3097 (69.10)	536 (17.31)	2561 (82.69)
Reimbursed for sterilization	1385 (30.90)	261 (18.84)	1124 (81.16)
*Age (years)*			
≤ 19	121 (2.70)	1 (0.83)	120 (99.17)
20–24	516 (11.51)	30 (5.81)	486 (94.19)
25–29	1107 (24.70)	170 (15.36)	937 (84.64)
30–34	1346 (30.03)	224 (16.64)	1122 (83.36)
35–49	1120 (24.99)	278 (24.82)	842 (75.18)
40–44	261 (5.82)	90 (34.48)	171 (65.52)
≥ 45	11 (0.25)	4 (36.36)	7 (63.64)
*Marital status*			
Married	3121 (69.67)	557 (17.85)	2564 (82.15)
Single	1176 (26.25)	181 (15.39)	995 (84.61)
Others/missing	183 (4.08)	59 (32.24)	124 (67.76)
*Parity*			
1	1990 (44.40)	19 (0.95)	1971 (99.05)
2	1522 (33.96)	394 (25.89)	1128 (74.11)
3	651 (14.52)	256 (39.32)	395 (60.68)
≥ 4	319 (7.12)	128 (40.13)	191 (59.87)
*Route of delivery*			
Vaginal delivery	2191 (48.94)	167 (7.62)	2024 (92.38)
Cesarean section	2286 (51.06)	630 (27.56)	1656 (72.44)
*Gestational age*			
22–28	65 (1.45)	5 (7.69)	60 (92.31)
> 28–34	232 (5.18)	40 (17.24)	192 (82.76)
> 34–36	417 (9.30)	96 (23.02)	321 (76.98)
> 36	3768 (84.07)	656 (17.41)	3112 (82.59)
*Birthweight*			
Very low birth weight (< 1500 g)	142 (3.17)	11 (7.75)	131 (92.25)
Low birth weight (1500–2500 g)	538 (12.00)	97 (18.03)	441 (81.97)
Normal birth weight (2500–4000 g)	3738 (83.40)	672 (17.98)	3066 (82.02)
High birth weight (> 4000 g)	64 (1.43)	17 (26.56)	47 (73.44)

The overall rate of immediate postpartum sterilization was 17.8%. Sterilization rate was highest in women who were hospital officers (23%), followed by self-paid women (20.2%); sterilization rate was about 17% for all other healthcare plans. When grouped into those who were reimbursed and not reimbursed for the procedure, the sterilization rate was similar, 17.3% and 18.8% respectively. Sterilization rate was highest in women aged 40–44 years (34.5%) and in multiparous women (1%, 25.9%, 39.3%, and 40.1% for women in their 1st, 2nd, 3rd and 4th pregnancies, respectively).

Table 2 shows sterilization rate of each reimbursement status. Sterilization uptake is similarly distributed in both groups when stratified by age, marital status, parity, and route of delivery. Table 3 shows the association between demographic factors and healthcare plans with sterilization. Women whose plans covered sterilization trended toward increased sterilization when compared with those who were not, but this was not statistically significant (OR = 1.11; 95% CI 0.94–1.31). Women aged 40–44 years were 8.53 times as likely to choose sterilization than those aged 20–24 years (OR = 8.53; 95% CI 5.45–13.35). Women who reported their status as married or single were equally likely to undergo sterilization (OR = 1.19; 95% CI 0.99–1.43). Women who delivered via cesarean section were more likely to undergo sterilization at the time of delivery (OR = 4.61; 95% CI 3.84–5.53).

**Table 2:** Sterilization uptake in each reimbursement status

	Not reimbursed		Reimbursed	
	Total	Sterilization rate	Total	Sterilization rate
*n (%)*	3097 (69.10)	536 (17.31)	1385 (30.90)	261 (18.84)
*Age (years)*				
≤ 19	38 (1.23)	–	83 (5.99)	1 (1.20)
20–24	316 (10.20)	14 (4.43)	200 (14.44)	16 (8.00)
25–29	786 (25.38)	114 (14.50)	321 (23.18)	56 (17.45)
30–34	946 (30.55)	152 (16.07)	400 (28.88)	72 (18.00)
35–49	821 (26.51)	192 (23.39)	299 (21.59)	86 (28.76)
40–44	182 (5.88)	61 (33.52)	79 (5.70)	29 (36.71)
≥ 45	8 (0.26)	3 (37.50)	3 (0.22)	1 (33.33)
*Marital status*				
Married	2260 (72.97)	385 (17.04)	861 (62.26)	172 (19.98)
Single	722 (23.31)	116 (16.07)	454 (32.83)	65 (14.32)
Others/missing	115 (3.71)	35 (30.43)	68 (4.92)	24 (35.29)
*Parity*				
1	1373 (44.33)	12 (0.87)	617 (44.55)	7 (1.13)
2	1083 (34.97)	279 (25.76)	439 (31.70)	115 (26.20)
3	440 (14.21)	173 (39.32)	211 (15.23)	83 (39.34)
≥ 4	201 (6.49)	72 (35.82)	118 (8.52)	56 (47.46)
*Route of delivery*				
Vaginal delivery	1520 (49.11)	107 (7.04)	671 (48.55)	60 (8.94)
Cesarean section	1575 (50.89)	429 (27.24)	711 (51.45)	201 (28.27)

**Table 3:** Odds ratio of postpartum sterilization

Factor	Postpartum sterilization		
	Crude OR (95% CI)	Adjusted OR^a^ (95% CI)	P-value
*Reimbursement*			
Not reimbursed for sterilization	Reference	Reference	Reference
Reimbursed for sterilization	1.11 (0.94, 1.31)	1.05 (0.86, 1.28)	0.632
*Health care coverage*			
Social security	0.81 (0.60, 1.10)		
Self-paid	Reference		
Universal coverage	0.92 (0.66, 1.28)		
Hospital officer	1.18 (0.72, 1.93)		
Government officer	0.82 (0.55, 1.22)		
*Age (years)*			
≤ 19	0.14 (0.02, 1.00)	0.28 (0.03, 2.20)	0.224
20–24	Reference	Reference	Reference
25–29	**2.94 (1.96, 4.40)**	**1.71 (1.09, 2.69)**	**0.019**
30–34	3.23 (2.18, 4.80)	1.49 (0.95, 2.32)	0.079
35–49	**5.35 (3.61, 7.92)**	**1.92 (1.24, 2.99)**	**0.004**
40–44	**8.53 (5.45, 13.35)**	**2.70 (1.61, 4.53)**	** < 0.001**
≥ 45	9.26 (2.57, 33.38)	1.53 (0.35, 6.72)	0.570
*Marital status*			
Married	1.19 (0.99, 1.43)	1.05 (0.85, 1.31)	0.642
Single	Reference	Reference	Reference
Others/missing	2.62 (1.85, 3.70)	1.23 (0.82, 1.86)	0.320
*Parity*			
1	**0.03 (0.02, 0.04)**	**0.03 (0.02, 0.04)**	** < 0.001**
2	Reference	Reference	Reference
3	**1.86 (1.53, 2.25)**	**2.08 (1.68, 2.58)**	** < 0.001**
4	**2.04 (1.53, 2.73)**	**2.59 (1.88, 3.58)**	** < 0.001**
≥ 5	**1.63 (1.05, 2.54)**	**2.65 (1.61, 4.38)**	** < 0.001**
*Route of delivery*			
Vaginal delivery	Reference	Reference	Reference
Cesarean section	**4.61 (3.84, 5.53)**	**5.87 (4.77, 7.24)**	** < 0.001**

Bold values indicate statistical significance

^a^Adjusted for age, parity, marital status, delivery route, and period of delivery (pre -vs during pandemic)

The following factors were found to be associated with increased utilization of female sterilization: age, parity, and delivery route. After adjusting for age, parity, marital status, delivery route, and period of delivery, reimbursement was not found to be associated with sterilization (adjusted OR (aOR) 1.05; 95% CI 0.86–1.28, p-value 0.632). Women aged 40–44 years old were 2.70 times more likely to undergo female sterilization (aOR = 2.70; 95% CI 1.61–4.53) than women aged 20–24 years old; whereas women aged 25–29 (aOR = 1.71; 95% CI 1.09–2.69) and 30–34 years old (aOR = 1.49; 95% CI 0.95–2.32) were approximately 1.5 times more likely to do so. When compared with women in their second pregnancy, those in their third, fourth, and fifth pregnancy had higher odds of choosing sterilization, for which the aOR were 2.08 (95% CI 1.68–2.58), 2.59 (95% CI 1.88–3.58), and 2.65 (95% CI 1.61–4.38), respectively. Women delivered via cesarean section were five times more likely to choose female sterilization compared to vaginal delivery (aOR = 5.87; 95% CI 4.77–7.24). The stratification of socioeconmic and clinical characteristics by COVID-19 pandemic is shown in Table 4. The rate of sterilization was comparable before and during the pandemic.

**Table 4:** Sterilization uptake before and during COVID-19 pandemic

Factor	Pre-COVID		During COVID	
	n (%)	Sterilization rate n (%)	n (%)	Sterilization rate n (%)
n (%)	2328 (100)	413 (17.74)	2154 (100)	384 (17.83)
*Health care coverage*				
Social security	1480 (63.57)	265 (17.91)	1330 (61.75)	213 (16.02)
Self-paid	135 (5.80)	22 (16.30)	152 (7.06)	36 (23.68)
Universal coverage	504 (21.65)	86 (17.06)	404 (18.76)	85 (21.04)
Hospital officer	51 (2.19)	14 (27.45)	88 (4.09)	18 (20.45)
Government officer	158 (6.79)	26 (16.46)	180 (8.36)	32 (17.78)
*Reimbursement*				
Not reimbursed for sterilization	1615 (69.37)	287 (17.77)	1482 (68.80)	249 (16.80)
Reimbursed for sterilization	713 (30.63)	126 (17.67)	672 (31.20)	135 (20.09)
*Age (years)*				
≤ 19	86 (3.69)	1 (1.16)	35 (1.62)	–
20–24	267 (11.47)	19 (7.12)	249 (11.56)	11 (4.42)
25–29	593 (25.47)	92 (15.51)	514 (23.86)	78 (15.18)
30–34	689 (29.60)	108 (15.67)	657 (30.50)	116 (17.66)
35–49	552 (23.71)	137 (24.82)	568 (26.37)	141 (24.82)
40–44	135 (5.80)	53 (39.26)	126 (5.85)	37 (29.37)
≥ 45	6 (0.26)	3 (50.00)	5 (0.23)	1 (20.00)
*Marital status*				
Married	1637 (70.35)	297 (18.14)	1484 (68.93)	260 (17.52)
Single	589 (25.31)	83 (14.09)	587 (27.26)	98 (16.70)
Others/missing	101 (4.34)	33 (32.67)	82 (3.81)	26 (31.71)
*Parity*				
1	1026 (44.07)	9 (0.88)	964 (44.75)	10 (1.04)
2	780 (33.51)	197 (25.26)	742 (34.45)	197 (26.55)
3	343 (14.73)	136 (39.65)	308 (14.30)	120 (38.96)
≥ 4	179 (7.69)	71 (39.66)	140 (6.50)	57 (40.71)
*Route of delivery*				
Vaginal delivery	1150 (49.44)	86 (7.48)	1041 (48.40)	81 (7.78)
Cesarean section	1176 (50.56)	327 (27.81)	1110 (51.60)	303 (27.30)
*Gestational age*				
22–28	37 (1.59)	2 (5.41)	28 (1.30)	3 (10.71)
> 28–34	121 (5.20)	24 (19.83)	111 (5.15)	16 (14.41)
> 34–36	235 (10.09)	56 (23.83)	182 (8.45)	40 (21.98)
> 36	1935 (83.12)	331 (17.11)	1833 (85.10)	325 (17.73)
*Birthweight*				
< 1500 g	76 (3.26)	4 (5.26)	66 (3.06)	7 (10.61)
1500–2500 g	276 (11.86)	52 (18.84)	262 (12.16)	45 (17.18)
2500–4000 g	1943 (83.46)	347 (17.86)	1795 (83.33)	325 (18.11)
> 4000 g	33 (1.42)	10 (30.30)	31 (1.44)	7 (22.58)

## Discussion

Our study found that the overall sterilization rate was 17.8%. Older age, parity, and delivery route via cesarean section were significantly associated with postpartum sterilization. We found that the reimbursement for the procedure was not significantly associated with postpartum sterilization, although women who were reimbursed for the procedure trended towards sterilization.

The sterilization rate in our study was lower than that previously published in Thailand. The prevalence of sterilization was reported to be 22% in 1969–1987 [11] and increased to as high as 33%–36% in the 1980s [8] following Thailand’s efforts to curb population growth by integrating contraception services into maternal and newborn care and by improving education and access to contraception [7]. Although the global prevalence of female sterilization is increasing, sterilization rates are decreasing in most countries [2] and our findings reflect this downward trend. Access to modern contraception such as contraceptive implants, IUDs, and injectable contraceptives coincides with reduction in sterilization rate, as reported in India [3] and the United Kingdom [12], and though data in East and Southeast Asia is sparce, this may explain our findings.

Sterilization rate in our study is relatively higher than in Western countries. We postulate that this is a contribution of both lower barriers to sterilization and lack of healthcare coverage in our country for modern contraceptive methods. Previous studies in the UK and US showed that barriers to sterilization such as mandatory waiting time, lack of operating room and anesthesiologist availability, and overly complicated consent process result in reduced sterilization rate and unfulfilled sterilization requests [9, 11–15]. The process for sterilization in our hospital does not include such barriers, allowing for easier access to postpartum sterilization. Studies in the UK and US have also observed that patients who were not covered for modern contraception were more likely to choose sterilization to avoid future costs [6]. At our hospital, other methods of contraception, such as COCs, are currently not covered by health plans and implants, and IUDs are reimbursed only to those under 20 years of age or with a Bangkok-registered address. Although patients in our hospital have access to modern contraceptive methods, a lack of insurance coverage may account for the relatively high sterilization rate.

Notably, we found that healthcare coverage did not affect postpartum sterilization rate. There was a trend toward increased sterilization in women whose plans covered sterilization compared to those whose plans did not, but this finding did not reach statistical significance. Other studies were conflicting; some have found that sterilization rate in privately versus publicly insured women do not differ [16, 17], while others [18, 19] have found that reimbursement plans affect sterilization rate. This disparity of findings may be because some insurance plans represent a tangle of multifactorial barriers to postpartum sterilization, for example the Medicaid sterilization policy in the US [4, 9, 17, 19]. In our setting, the difference in healthcare plans for postpartum sterilization reflects only reimbursement for the procedure because the pre-procedure counseling and informed consent process is similar for all plans, and all plans similarly do not cover other contraceptive options.

We found that demographic factors associated with postpartum sterilization included older age, parity, and delivery route which is in accordance with other studies where older women with more children were more likely to opt for postpartum and interval sterilization [3, 20–22]. Our hospital offers sterilization to all women after thorough counseling and has no policy to deny sterilization based on demographic factors. However, women in their third (or more) pregnancy are counseled that sterilization as a strong option if they do not desire more children, which may account for our findings.

Our study found that women with cesarean deliveries were more likely to undergo sterilization than those who delivered vaginally, which is consistent with previous studies [23, 24]. Postpartum sterilization after vaginal delivery requires a second, separate procedure as opposed to cesarean delivery where sterilization can be performed in a single operation. This poses a barrier to sterilization in women who delivered vaginally because women may be reluctant to undergo a second procedure and due to barriers associated with scheduling a second operation such as operating room availability [25]. As our hospital is a tertiary hospital and a referral center for obstetric cases, we have a relatively high cesarean delivery rate which may account for the higher sterilization rate. The desire to avoid repeat cesarean delivery may also account for higher sterilization rate in abdominal deliveries.

The major strength of this study is that it is analysis from a large data set including all women who delivered over a 12 month period. Our study was also conducted in a setting with minimal barriers to immediate postpartum sterilization. Multivariable analysis was used to adjust for potential confounders, strengthening our findings. This study had several limitations, the first of which is its secondary nature. The original study was designed to examine the effects of the pandemic on postpartum contraceptive choice; however, we found that COVID-19 did not affect the decision to initiate postpartum contraception. Another limitation was that we did not collect data on the days of the week that postpartum sterilization was requested and were unable to comment on how this affects sterilization rates, as those who delivered vaginally on Friday would have to wait until Monday to undergo the operation.

Further studies are required to determine unfulfilled sterilization requests in our hospital setting. The effects of repeated cesarean sections on sterilization, sterilization regret, and vasectomy also warrant further investigation. Male involvement in postpartum sterilization decision-making needs further study.

Defining the population most likely to undergo female postpartum sterilization can inform actions to better meet the demands for sterilization. Older women with more children are more likely to undergo sterilization, as are women who deliver via cesarean section. Healthcare coverage plans did not affect the use of postpartum sterilization in our setting.

## Data Availability

Not applicable.
